# Ensemble machine learning models for predicting bone metastasis in bladder cancer

**DOI:** 10.3389/fonc.2025.1653506

**Published:** 2025-09-25

**Authors:** Zhan Jiang Yu, Xiang Da Xu, Xin Chang Zou, Pei De Su, Hai Chao Chao, Tao Zeng

**Affiliations:** ^1^ The Second Affiliated Hospital, Jiangxi Medical College, Nanchang University, Nanchang, China; ^2^ Department of Urology, Second Affiliated Hospital of Nanchang University, Nanchang, China

**Keywords:** machine learning, bladder cancer, SEER database, bone metastasis, predictive value

## Abstract

**Background and purpose:**

The occurrence of bone metastasis (BM) in advanced bladder cancer (BC) often signifies a poor prognosis. Currently, the accurate prediction of BM in BC remains a challenge. This study develops predictive models using machine learning algorithms to predict bladder cancer bone metastasis (BCBM) and aid in personalized clinical decisions.

**Patients and methods:**

We reviewed and analyzed data from patients diagnosed with BC between 2010 and 2015 in the Surveillance, Epidemiology, and End Results (SEER) database. In addition, we included 327 patients treated at the Second Affiliated Hospital of Nanchang University and Jiangxi Cancer Hospital as an external validation cohort. Independent risk factors for BM in patients with BC were identified through univariate and multivariate logistic regression analyses. These features were then integrated into seven machine learning algorithms to build predictive models: logistic regression (LR), support vector machine (SVM), gradient boosting machine (GBM), neural network (NN), random forest (RF), extreme gradient boosting (XGB), and *k*-nearest neighbors (KNN). The performance of these models was evaluated using the area under the receiver operating characteristic curve (AUC), along with accuracy, sensitivity (recall), and specificity.

**Results:**

A total of 22,114 patients diagnosed with BC were included in this study, with 537 (2.4%) patients developing BM. The identified independent risk factors for BCBM included age, race, tumor histology, tumor grade, T stage, N stage, the presence of brain metastasis, liver metastasis, and lung metastasis, and history of radiotherapy. Among the seven developed machine learning models, the tree-based GBM model exhibited the best performance in the test set, achieving AUC, accuracy, sensitivity, and specificity values of 0.855, 0.813, 0.733, and 0.815, respectively. The GBM model also demonstrated robust performance in the external validation set, achieving an AUC of 0.766 and accuracy of 0.945. According to Shapley additive explanations (SHAP), the most significant feature in the GBM prediction model is the T stage, followed by the N stage and radiotherapy.

**Conclusion:**

The GBM model offers a precise and personalized approach to predicting BCBM, potentially enhancing clinical decision-making and the efficiency of BM screening in patients with BC.

## Introduction

Bladder cancer (BC) is the second most common urogenital cancer (1). Worldwide, it ranks as the ninth most prevalent cancer, with approximately 614,000 new cases and 220,000 deaths reported in 2022 (2). BC is characterized by a high rate of recurrence and metastasis (3). Metastatic bladder cancer (mBC) primarily spreads to the lymph nodes, the bones, the lungs, and the liver (4). Approximately 10%–15% of patients with BC are diagnosed with metastasis at the initial presentation (5), with the bone being the most common site of metastasis (6, 7). Bone metastasis (BM) can lead to skeletal-related events (SREs), which often result in complications such as pain, hypercalcemia, spinal cord compression, pathological fractures, and neurological deficits. These complications significantly diminish the patient’s quality of life (8) and adversely affect survival rates (9), with the 1-year survival rate for patients with bladder cancer bone metastasis (BCBM) as low as 21% (10). The TNM staging system established by the American Joint Cancer Committee (AJCC) is widely recognized for predicting the metastasis risk and the prognosis of various cancer patients (11). However, the TNM system does not account for additional risk factors such as age, gender, and previous treatment history, which have been shown to be valuable in predicting BC metastasis (12, 13). Consequently, the predictive accuracy of the TNM staging system for patients with BM may be limited. Many patients with BC may not receive a timely diagnosis of BM, potentially missing optimal treatment windows and leading to poorer prognosis. Therefore, accurately predicting the occurrence of BM in patients with BC is of great significance.

In recent years, artificial intelligence (AI) models based on machine learning (ML) algorithms have been increasingly integrated into clinical practice (14, 15). As a key branch of AI, ML has been utilized to independently extract features from large datasets and construct high-precision prediction models, continuously optimizing the performance of these algorithms.

In medical research, the construction and validation of models based on ML can uncover potential patterns in large clinical datasets, providing valuable tools for early diagnosis and prognosis assessment. ML has been widely applied in the prognostic evaluation of prostate cancer, kidney cancer, and gastrointestinal cancer, as well as in studies of organ metastasis (16, 17). The rapid advancement of health big data in biomedical science has revealed the significant potential of ML applications in understanding disease and in health management (18).

Currently, there are limited studies exploring ML models for the prediction of BCBM. In this study, we evaluated seven ML algorithms and observed that, among them, the gradient boosting machine (GBM) model showed relatively better performance. This study extracted data on patients with BC, as well as their clinical and pathological characteristics, from the Surveillance, Epidemiology, and End Results (SEER) database for the years 2010–2015. Accurate and reliable ML models to predict BCBM were constructed, which could assist clinicians in promptly identifying patients with BM. This approach aims to provide personalized clinical strategies for patients and promote the rational allocation of medical resources.

## Methods

### Ethics statement

The SEER database is a publicly available, anonymized cancer registry where all patient data have been de-identified. Therefore, this study was exempt from ethics review and patient consent requirements.

### Patient selection and variables

**Figure 1:**
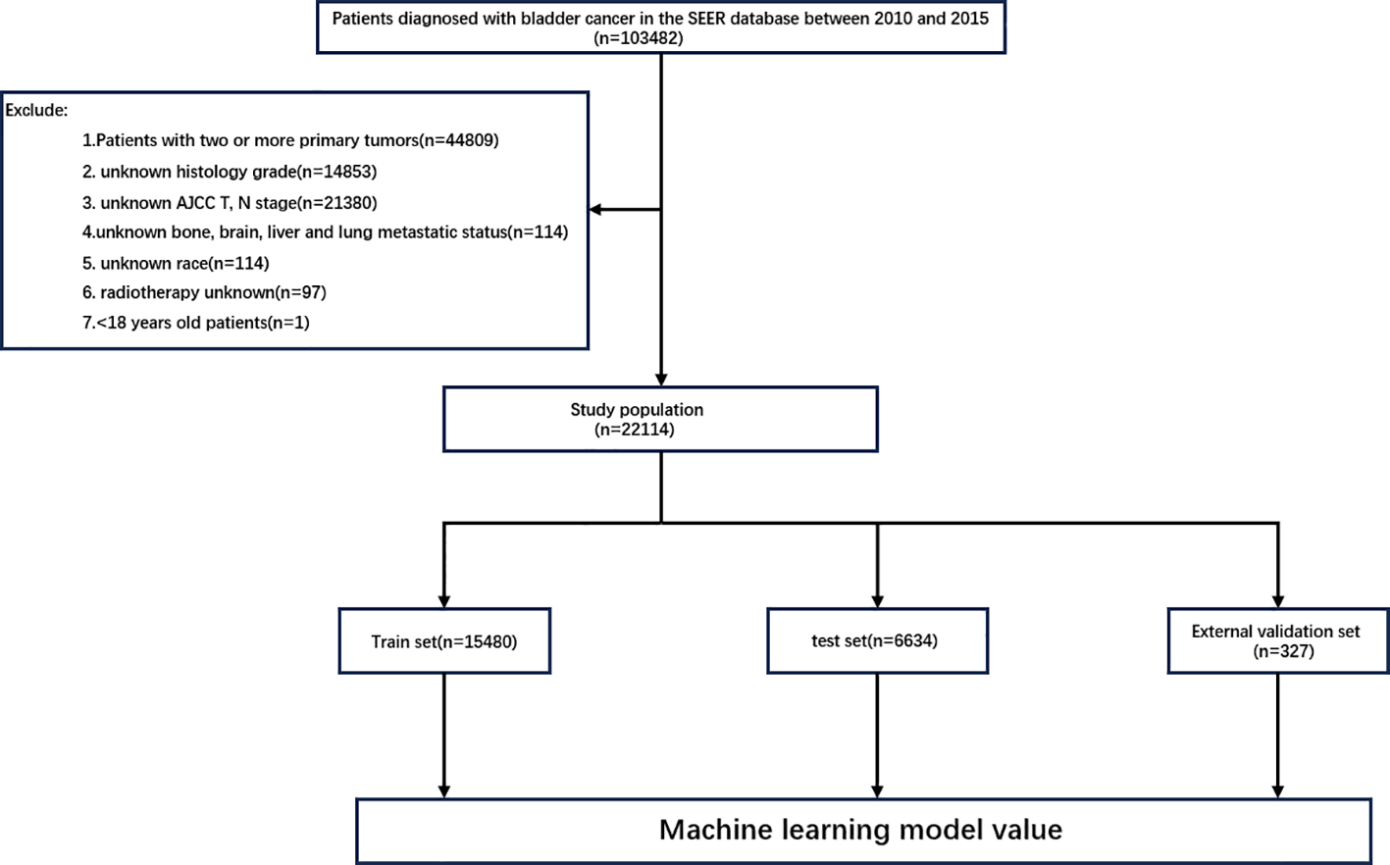
Study flowchart of case screening.

All data were extracted from the SEER database using SEERStat software (version 8.4.4). This database covers approximately 28% of the US population and includes 17 population-based cancer registries, providing clinicopathological, demographic, and survival outcome information. The case listing was based on the dataset of Incidence—SEER Research Data, 17 Registries, Nov 2023 Sub (2000–2021). Subjects with BC were identified using site codes C67.0–C67.9. In this study, patients with a diagnosis of malignant BC by positive histology diagnosed between 2010 and 2015 were selected. The exclusion criteria were as follows: 1) patients under the age of 18 years; 2) patients with unknown AJCC T or N staging; 3) patients with unknown race or histological grade; 4) patients with unknown bone, brain, liver, or lung metastasis status; 5) patients with unknown radiotherapy or chemotherapy information; and 6) patients with two or more primary tumors. The flowchart for the case screening is shown in **Figure 1**. The external validation cohort comprised 327 patients with pathologically confirmed BC diagnosed between 2016 and 2023, among whom 11 developed BM. The final follow-up was completed in November 2024. This study was approved by the Institutional Review Boards of the Second Affiliated Hospital of Nanchang University and Jiangxi Cancer Hospital, with a waiver of informed consent granted. A total of 13 variables related to patient demographics and clinicopathological characteristics were extracted for analysis. The demographic variables included age, sex, and race, while the clinicopathological variables included tumor histology type, tumor grade, T stage, N stage, radiotherapy, chemotherapy, brain metastasis, BM, lung metastasis, and liver metastasis. Patient age was categorized into three subgroups, <60 years, 60–80 years, and >80 years, and the tumor grade into two subgroups. The histological types were classified into transitional cell carcinoma, squamous cell carcinoma, adenocarcinoma, and other types. All cancer patients exhibited histopathological and morphological evidence consistent with the International Classification of Diseases for Oncology, Third Edition (ICD-O-3), and all BC patients were staged according to the AJCC 7th Edition guidelines and the SEER staging information.

### Data processing and feature engineering

All statistical analyses and data descriptions were conducted using R version 4.4.1 and SPSS version 27. The continuous variable age was converted into a categorical variable, which was then processed using the label encoding method. In this study, logistic regression analysis was performed on the variables collected from the SEER database using R software to identify features suitable for ML models. Significant variables in patients with BCBM were identified through univariate logistic regression analysis (*p* < 0.05). These variables were subsequently included in a multifactorial logistic regression analysis, and the ML models were built using the variables that remained statistically significant (*p* < 0.05) in the multivariate analysis. Correlation analysis was conducted to examine the relationships between the selected variables. In addition, to compare the importance of each feature, the feature importance in the ML model was extracted based on the principle of permutation importance. Finally, the importance of each feature was ranked using Shapley additive explanations (SHAP), helping decision-makers understand how to effectively utilize the model and comprehend the impact of each feature on the final predicted outcome. To achieve this, SHAP was employed to quantify the contribution of each feature to the model predictions, providing a transparent and interpretable analysis. Given that this dataset is unbalanced, which may affect the model performance, the synthetic minority oversampling technique (SMOTE) was employed as the sampling method in the training set to mitigate the impact of sample imbalance on the evaluation results.

### Model construction and evaluation

The data from the SEER database were randomly divided into a training set and a test set at a ratio of 7:3. In this study, seven ML algorithms were selected, including three tree-based models [random forest (RF), GBM, and extreme gradient boosting (XGB)]; a linear model (logistic regression, LR); a kernel-based model (support vector machine, SVM); a distance-based model (*k*-nearest neighbors, KNN); and neural networks. External validation was subsequently conducted to further evaluate the generalizability of the model. The evaluation indicators for the ML algorithms included the area under the receiver operating characteristic curve (AUC), accuracy, sensitivity, and specificity. The ML models were developed using the caret framework in R software. The relevant parameters of the model can be found in **Supplementary Table S1**.

## Results

### Patient characteristics and metastasis

A total of 22,114 patients with BC were included in this study. At the time of initial diagnosis, 21,577 patients (97.6%) had no BM, while 537 patients (2.4%) had BM. The patients were randomly divided into a training set (*n* = 15,480) and a test set (*n* = 6,634) at a 7:3 ratio. In the external validation cohort, 316 patients (96.6%) showed no evidence of BM, while 11 patients (3.4%) developed BM. The characteristics of all cohorts are presented in **Tables 1** and **2**.

**Table 1:** Clinical and pathological characteristics of the training and test sets.

Variable	Training set, *n* (%)		Test set, *n* (%)	
	NBM (*n* = 15,104)	BM (*n* = 376)	NBM (*n* = 6,473)	BM (*n* = 161)
Age (years)				
<60	2,585 (17.1)	98 (26.1)	1,133 (17.5)	48 (29.8)
60–80	8,533 (56.5)	222 (59.0)	3,677 (56.8)	84 (52.2)
>80	3,986 (26.4)	56 (14.9)	1,663 (25.7)	29 (18.0)
Sex				
Men	10,995 (72.8)	289 (76.9)	4,754 (73.4)	127 (78.9)
Women	4,109 (27.2)	87 (23.1)	1,719 (26.6)	34 (21.1)
Race				
White	13,145 (87.0)	315 (83.8)	5,697 (88.0)	134 (83.2)
Black	1,017 (6.7)	45 (12.0)	446 (6.9)	18 (11.2)
Other	942 (6.2)	16 (4.3)	330 (5.1)	9 (5.6)
Grade				
I–II	1,940 (12.8)	16 (4.3)	822 (12.7)	9 (5.6)
III–IV	13,164 (87.2)	360 (95.7)	5,651 (87.3)	152 (94.4)
Histologic type				
Transitional cell carcinoma	14,156 (93.7)	334 (88.8)	6,041 (93.3)	144 (89.4)
Squamous cell carcinoma	344 (2.3)	6 (1.6)	176 (2.7)	7 (4.3)
Adenocarcinoma	268 (1.8)	8 (2.1)	106 (1.6)	1 (0.6)
Other	336 (2.2)	28 (7.4)	150 (2.3)	9 (5.6)
T stage				
T1	7,916 (52.4)	63 (16.8)	3,351 (51.8)	35 (21.7)
T2	4,782 (31.7)	206 (54.8)	2,089 (32.3)	84 (52.2)
T3	1,390 (9.2)	32 (8.5)	593 (9.2)	11 (6.8)
T4	1,016 (6.7)	75 (19.9)	440 (6.8)	31 (19.3)
N stage				
N0	13,683 (90.6)	244 (64.9)	5,853 (90.4)	94 (58.4)
N1	559 (3.7)	37 (9.8)	248 (3.8)	21 (13.0)
N2	685 (4.5)	66 (17.6)	300 (4.6)	32 (19.9)
N3	177 (1.2)	29 (7.7)	72 (1.1)	14 (8.7)
Brain metastasis				
Yes	16 (0.1)	13 (3.5)	12 (0.2)	2 (1.2)
No	15,088 (99.9)	363 (96.5)	6,461 (99.8)	159 (98.8)
Liver metastasis				
Yes	161 (1.1)	70 (18.6)	56 (0.9)	33 (20.5)
No	14,943 (98.9)	306 (81.4)	6,417 (99.1)	128 (79.5)
Lung metastasis				
Yes	283 (1.9)	88 (23.4)	117 (1.8)	50 (31.1)
No	14,821 (98.1)	288 (76.6)	6,356 (98.2)	111 (68.9)
Radiotherapy				
Yes	1,520 (10.1)	119 (31.6)	674 (10.4)	43 (26.7)
No	13,584 (89.9)	257 (68.4)	5,799 (89.6)	118 (73.3)
Chemotherapy				
Yes	5,300 (35.1)	198 (52.7)	2,278 (35.2)	89 (55.3)
No	9,804 (64.9)	178 (47.3)	4,195 (64.8)	72 (44.7)

*NBM*, no bone metastasis; *BM*, bone metastasis.

**Table 2:** Clinical and pathological characteristics of the external validation set.

Variable, *n* (%)	External validation set (*n* = 327)
Age (years)	
<60	56 (17.1)
60–80	196 (59.9)
>80	75 (23.0)
Sex	
Men	91 (27.8)
Women	236 (72.2)
Race	
White	0
Black	0
Other	327 (100)
Grade	
I–II	33 (10.1)
III–IV	294 (89.9)
Histologic type	
Transitional cell carcinoma	303 (92.7)
Squamous cell carcinoma	11 (3.4)
Adenocarcinoma	6 (1.8)
Other	7 (2.1)
T stage	
T1	155 (47.4)
T2	110 (33.6)
T3	36 (11.1)
T4	26 (7.9)
N stage	
N0	287 (87.8)
N1	11 (3.4)
N2	27 (8.2)
N3	2 (0.6)
Brain metastasis	
Yes	2 (0.6)
No	325 (99.4)
Liver metastasis	
Yes	6 (1.8)
No	321 (98.2)
Lung metastasis	
Yes	5 (1.5)
No	322 (98.5)
Radiotherapy	
Yes	32 (9.8)
No	295 (90.2)
Chemotherapy	
Yes	125 (38.2)
No	202 (61.8)

### Feature filter

A total of 10 independent risk factors related to BM were identified through univariate and multivariate logistic regression analyses. These included age, race, tumor histology, tumor grade, T stage, N stage, radiotherapy, brain metastasis, lung metastasis, and liver metastasis (*p* < 0.05) (**Table 3**). Among these, the three most significant risk factors were brain metastasis (OR = 5.98, 95%CI = 2.37–15.14), liver metastasis (OR = 5.89, 95%CI = 4.05–8.56), and lung metastasis (OR = 5.87, 95%CI = 4.25–8.09). Based on these features, seven different models were developed in this study using ML algorithms.

**Table 3:** Univariate and multivariate logistic regression analyses of the variables.

Variable	Univariate analysis		Multivariate analysis	
	OR (95%CI)	*p*	OR (95%CI)	*p*
Age (years)				
<60	Reference		Reference	
60–80	0.69 (0.54–0.87)	0.002	0.75 (0.57–0.99)	0.041
>80	0.37 (0.27–0.52)	<.001	0.42 (0.29–0.61)	<0.001
Sex				
Women	Reference			
Men	1.24 (0.97–1.58)	0.080		
Race				
White	Reference		Reference	
Black	1.85 (1.34–2.54)	<0.001	1.45 (1.03–2.06)	0.035
Other	0.71 (0.43–1.18)	0.183	0.80 (0.47–1.37)	0.418
Grade				
I–II	Reference		Reference	
III–IV	3.32 (2.01–5.48)	<0.001	1.79 (1.04–3.09)	0.037
Histologic type				
Transitional cell carcinoma	Reference		Reference	
Squamous cell carcinoma	0.74 (0.33–1.67)	0.467	0.54 (0.23–1.28)	0.163
Adenocarcinoma	1.27 (0.62–2.58)	0.517	0.99 (0.46–2.11)	0.979
Other	3.53 (2.37–5.27)	<0.001	1.61 (1.01–2.57)	0.043
T stage				
T1	Reference		Reference	
T2	5.41 (4.07–7.19)	<0.001	2.91 (2.13–3.98)	<0.001
T3	2.89 (1.88–4.44)	<0.001	1.39 (0.87–2.23)	0.165
T4	9.28 (6.59–13.05)	<0.001	3.46 (2.32–5.15)	<0.001
N stage				
N0	Reference		Reference	
N1	3.71 (2.60–5.30)	<0.001	1.77 (1.17–2.67)	0.006
N2	5.40 (4.07–7.17)	<0.001	2.28 (1.62–3.21)	<0.001
N3	9.19 (6.08–13.88)	<0.001	4.33 (2.68–6.97)	<0.001
Brain metastasis				
No	Reference		Reference	
Yes	33.77 (16.13–70.73)	<0.001	5.98 (2.37–15.14)	<0.001
Liver metastasis				
No	Reference		Reference	
Yes	21.23 (15.69–28.73)	<0.001	5.89 (4.05–8.56)	<0.001
Lung metastasis				
No	Reference		Reference	
Yes	16.00 (12.26–20.88)	<0.001	5.87 (4.25–8.09)	<0.001
Radiotherapy				
No	Reference		Reference	
Yes	4.14 (3.31–5.18)	<0.001	3.08 (2.38–4.00)	<0.001
Chemotherapy				
No	Reference		Reference	
Yes	2.06 (1.68–2.53)	<0.001	0.94 (0.74–1.20)	0.643

*OR*, odds ratio; *95%CI*, 95% confidence interval.

### Importance of correlation analysis and features for prediction

**Figure 2:**
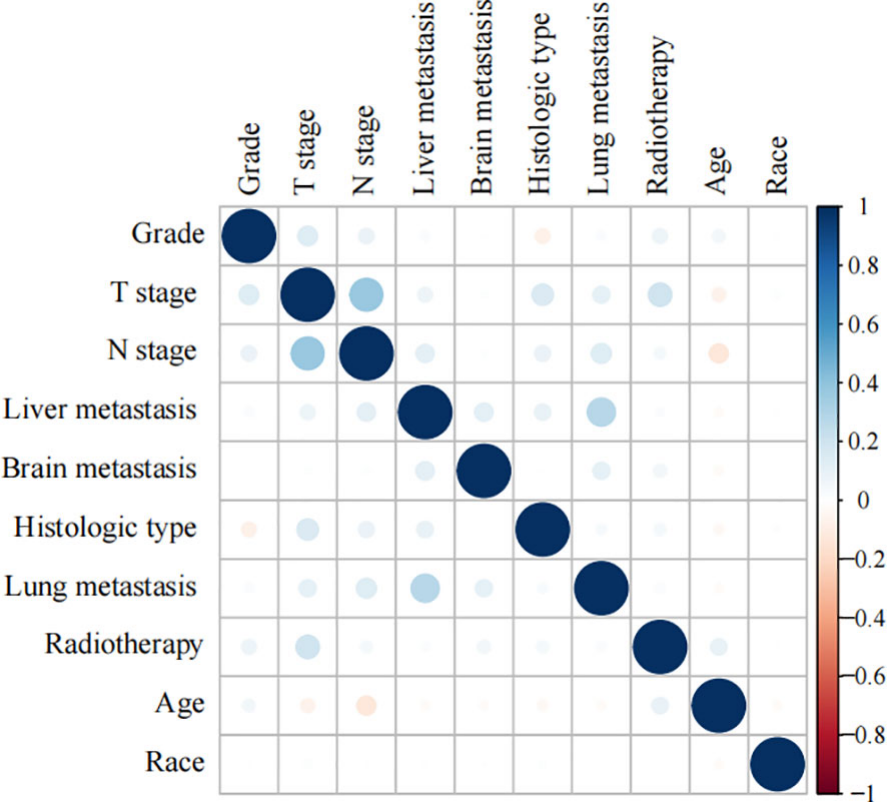
Heat map of the correlation of features.

**Figure 3:**
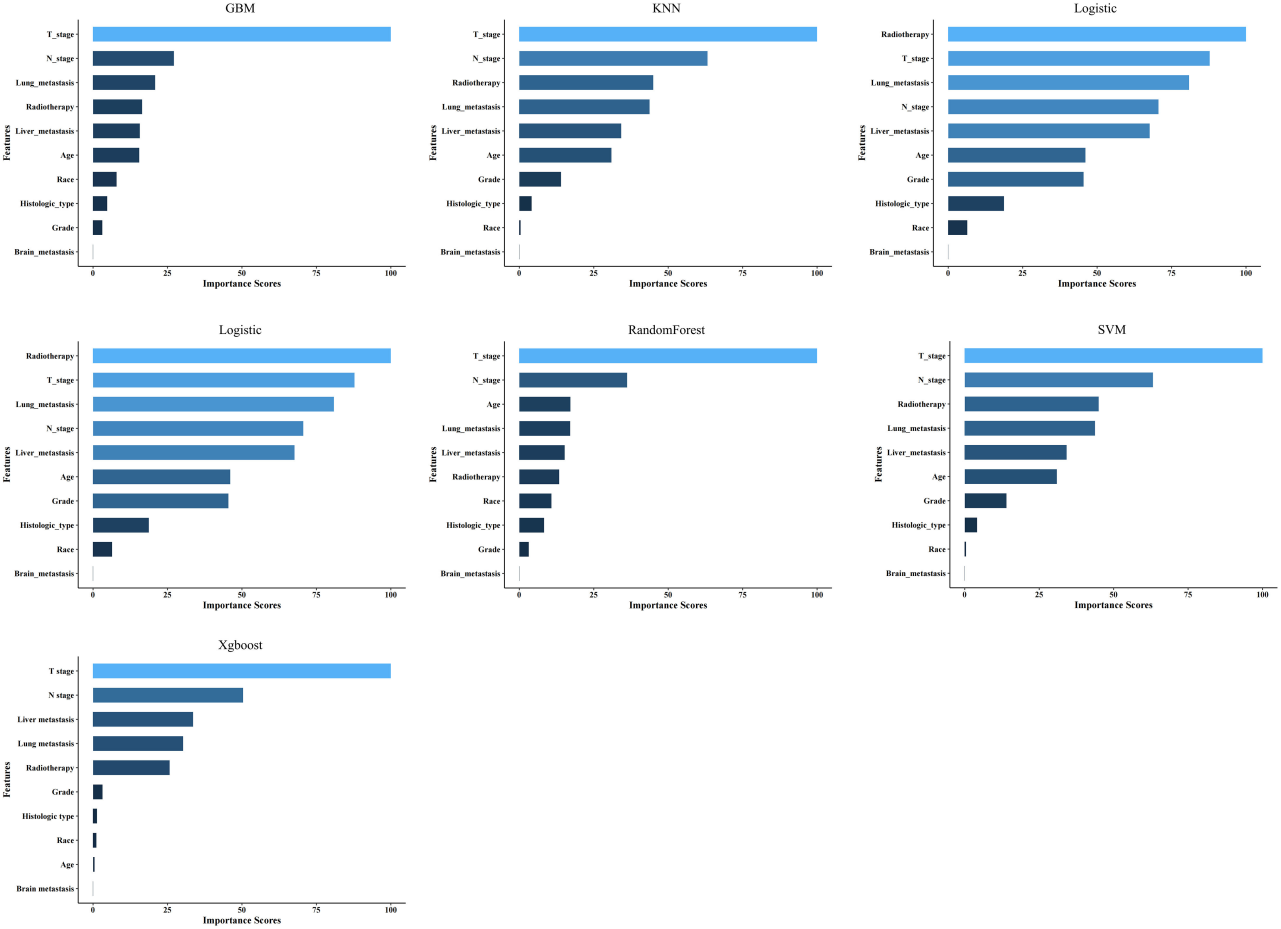
Feature importance of the different models.

**Figure 4:**
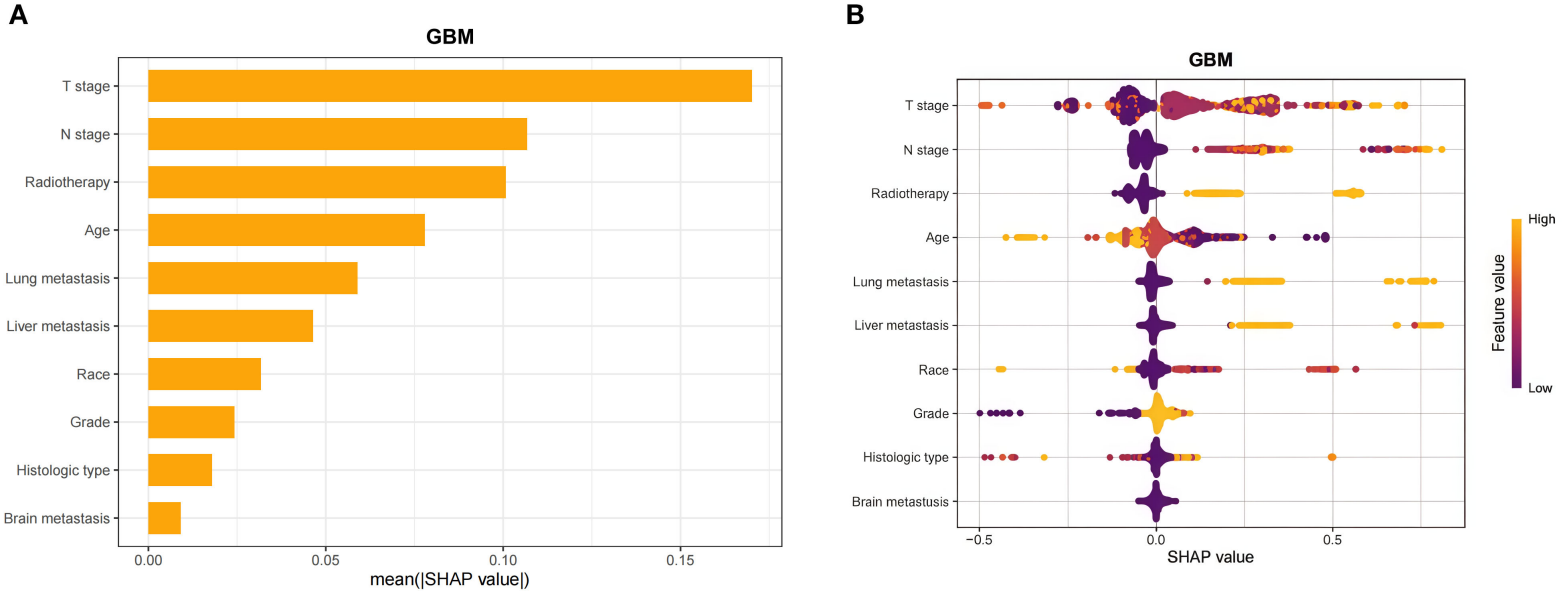
Interpretability of the gradient boosting machine (GBM) model assessed using the SHAP method. **(A)** SHAP bar chart showing the importance of each feature based on the mean SHAP values. **(B)** SHAP summary plot showing the impact of each feature on the model predictions. *Individual dots* symbolize patients, and *different colors* represent different levels of influence on the model output. *SHAP*, Shapley additive explanations.

Spearman’s correlation analysis was used to evaluate the correlation between factors and examine the independence of the data characteristics. As shown in **Figure 2**, the correlation heatmap illustrates no significant correlation among the 10 variables filtered using logistic regression. **Figure 3** displays the importance of the features extracted from the different ML algorithms. Notably, in the majority of the predictive models, T stage consistently emerged as the most influential feature, underscoring its critical role in predicting BM in BC. In contrast, tumor histology, tumor grade, race, and brain metastasis contributed relatively little to the model across most algorithms, with no significant differences in their importance. In the GBM model, the features ranked from the highest to the lowest importance were: T stage, N stage, lung metastasis, radiotherapy, liver metastasis, age, race, tumor histology, tumor grade, and brain metastasis. The SHAP values were then calculated for each variable in the GBM model, with the SHAP bar graph (**Figure 4A**) illustrating the importance of each feature. The results indicated that T stage, N stage, and radiotherapy are the most significant contributors to the GBM model. Both methods were consistent in identifying T stage and N stage as the top two characteristics, while the bottom four—race, tumor histology, tumor grade, and brain metastasis—were also nearly identical. A summary plot of the SHAP values is presented in **Figure 4B**, which explains the impact of each feature on the model predictions.

### Model performance and subgroup analysis

**Figure 5:**
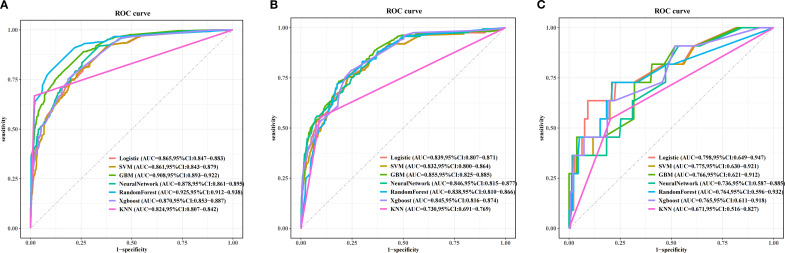
Receiver operating characteristic (ROC) curves of the prognostic models based on machine learning in the training set **(A)**, test set **(B)**, and the external validation set **(C)**.

**Figure 6:**
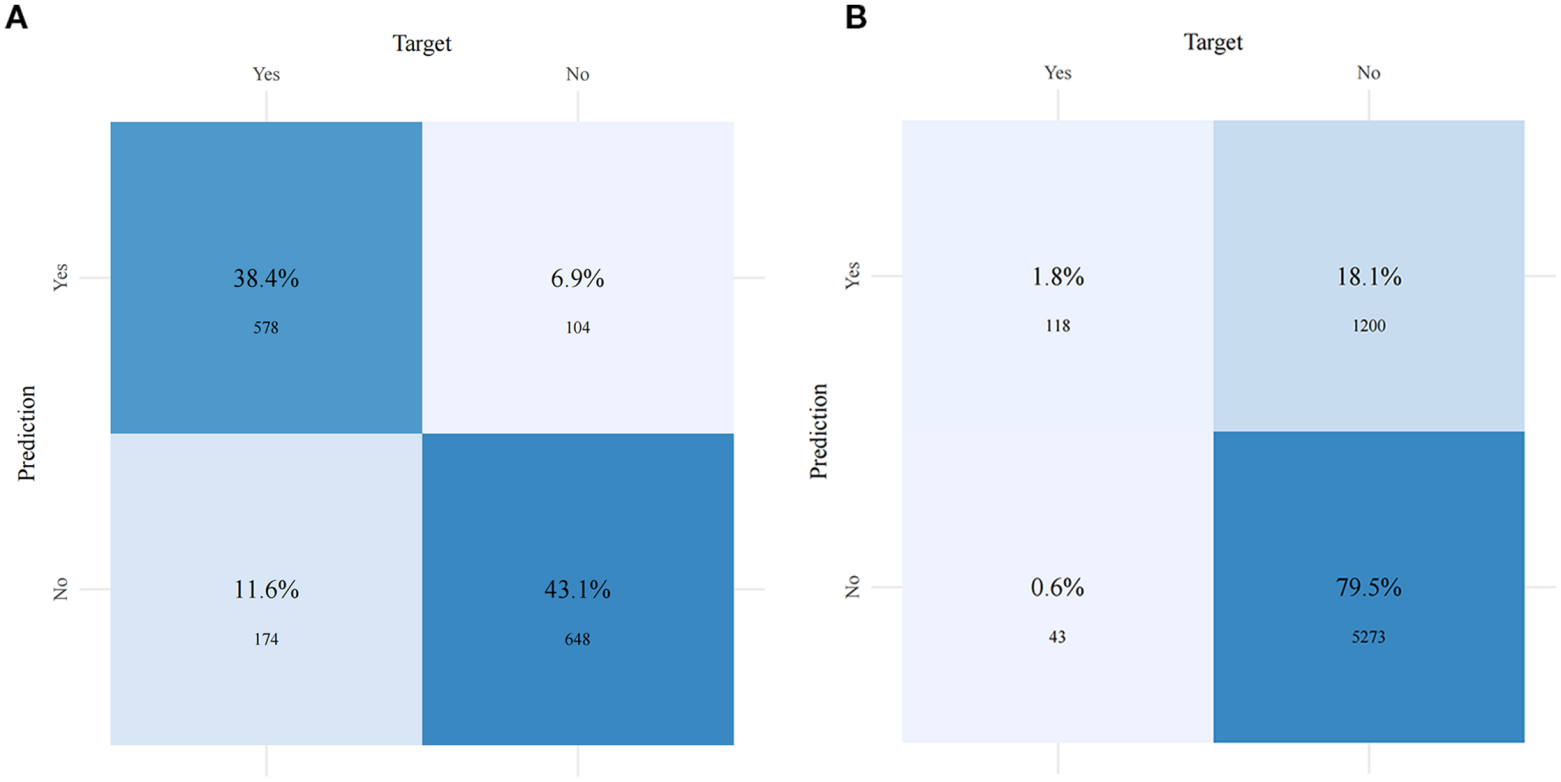
Confusion matrices of the gradient boosting machine (GBM) model in the training set **(A)** and the test set **(B)**.

**Figure 7:**
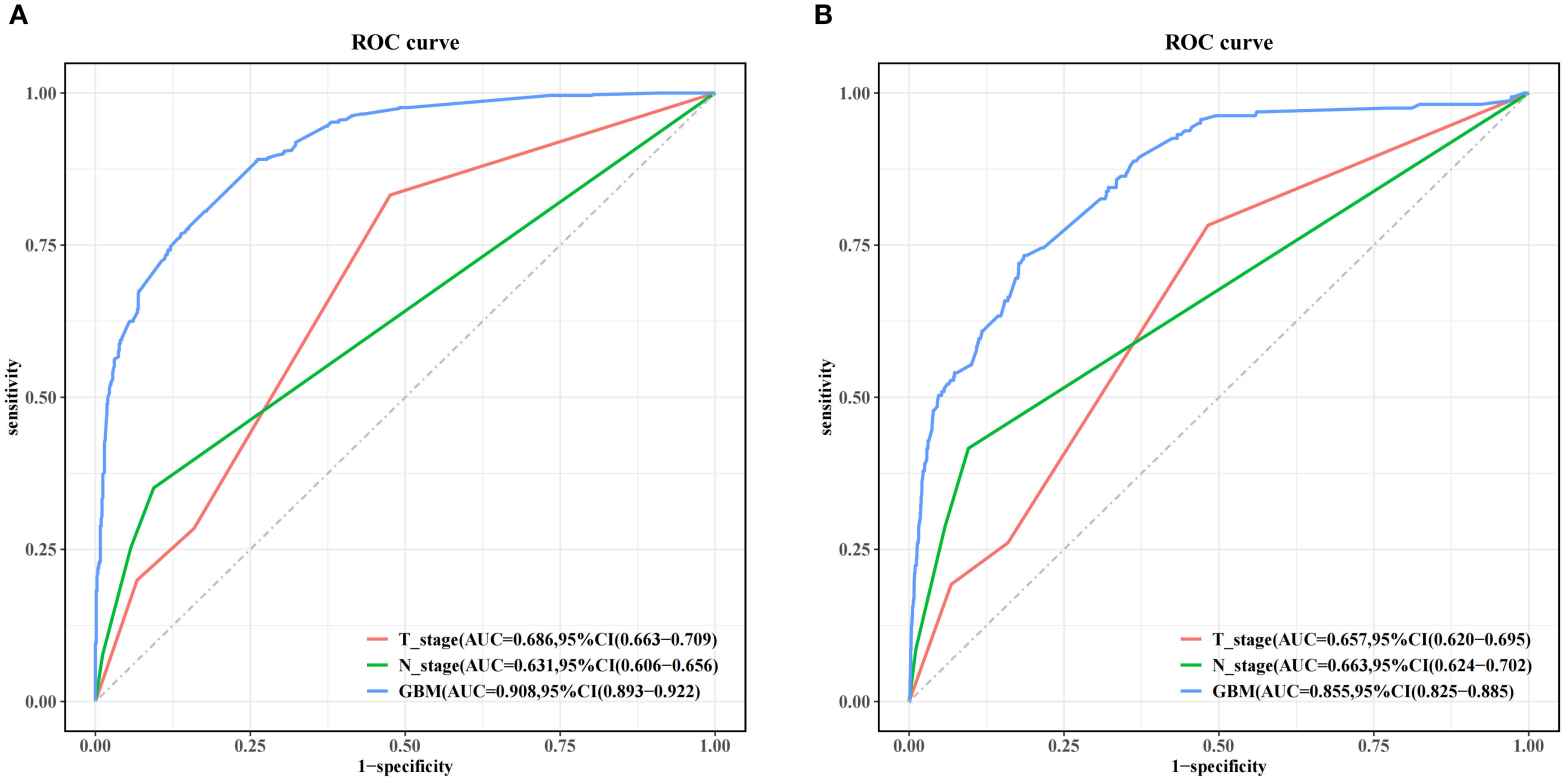
Performance comparison between the gradient boosting machine (GBM) model and TNM staging alone in both the training set **(A)** and the test set **(B)**.

**Figure 8:**
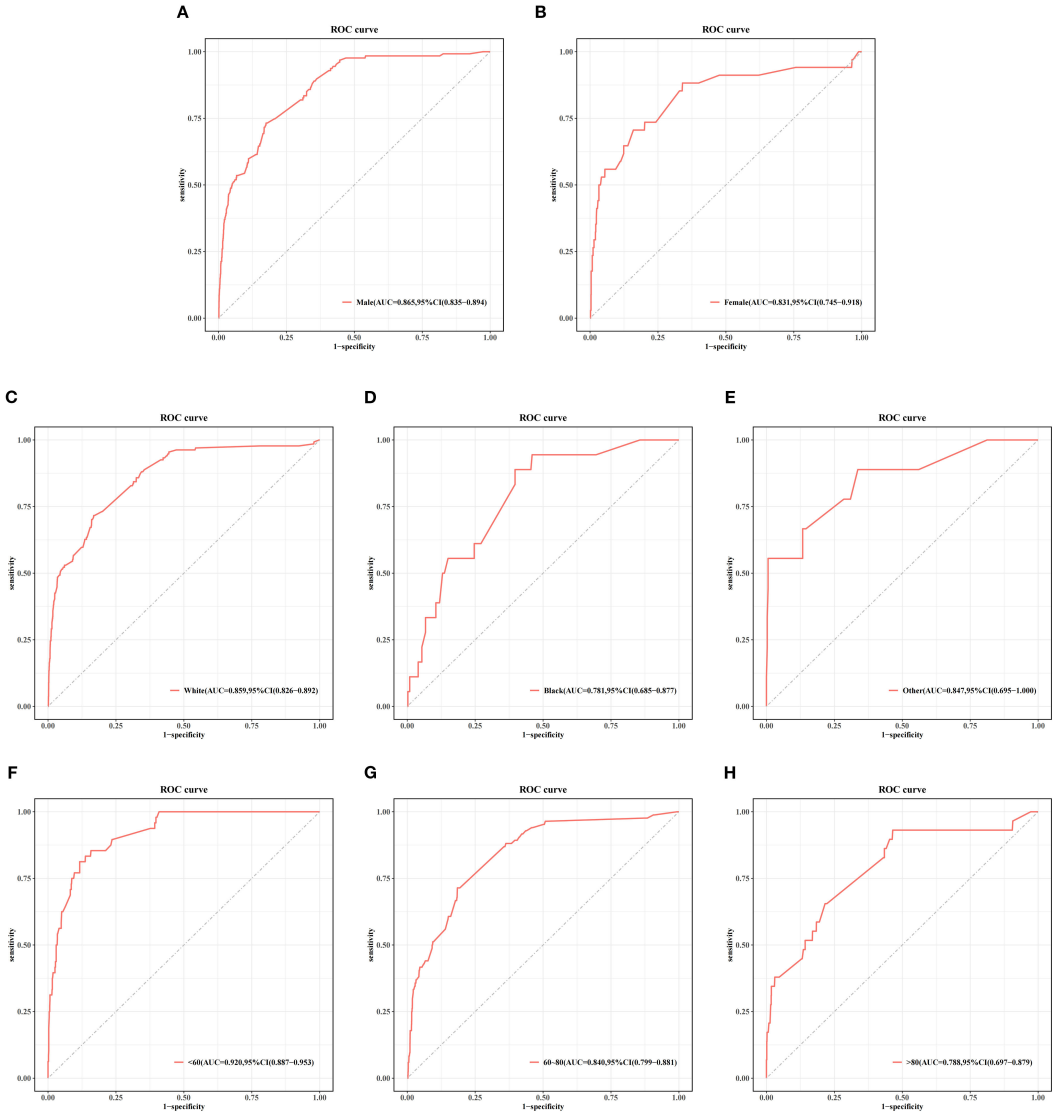
Stratified analysis of the gradient boosting machine (GBM) model performance by gender **(A, B)**, race **(C–E)**, and age **(F–H)** subgroups in the test set.

**Figure 5** and **Table 4** present the performance of the seven prediction models. The training set, balanced using SMOTE, was employed to train the models, while the test set was used to evaluate the accuracy and generalization ability of the models. To further validate the generalizability of the GBM model, external validation was performed using an independent cohort. Seven ML models were developed using the identified risk factors. After a comprehensive comparison, the GBM model demonstrated the best predictive value, achieving the highest AUC value of 0.855, along with accuracy, sensitivity (recall), and specificity values of 0.813, 0.733, and 0.815, respectively. The GBM model demonstrated favorable performance in the external validation cohort, achieving an AUC of 0.766 and an accuracy of 0.945 (**Supplementary Table S2**). The discrepancy between the model accuracy and AUC may be attributed to sample imbalance. Given that only 11 cases of BM were available, the model likely exhibited bias toward the majority class. This results in superficially high accuracy while limiting the model’s ability to identify minority class samples, consequently compromising the AUC performance. The confusion matrices for the GBM model in both the training and test sets are displayed in **Figure 6**. The predictive performance of the GBM model was compared with that of TNM staging to evaluate whether the model could provide more accurate and clinically meaningful predictions. As shown in **Figure 7**, the GBM model demonstrated superior performance to TNM staging alone, achieving an AUC of 0.855 compared with the lower AUC of TNM staging. This suggests that the GBM model may better capture features associated with the risk of BM. Stratified analyses of the model predictions were conducted to evaluate its fairness across demographic subgroups (**Figure 8**). Patients were stratified by gender, race, and age, with the model performance metrics calculated separately for each subgroup. The results showed comparable predictive performance between genders (AUC of 0.865 for male *vs*. 0.831 for female patients). Racial subgroup analysis revealed AUCs of 0.859 (white), 0.781 (black), and 0.847 (other). Age-stratified performance demonstrated AUCs of 0.920 (<60 years), 0.840 (60–80 years), and 0.788 (>80 years). While some inter-subgroup variability was observed, the model maintained clinically acceptable performance across all demographic strata.

**Table 4:** Test set predictive performance of the different models.

Model	AUC	Accuracy	Sensitivity	Specificity
LR	0.839	0.764	0.764	0.764
SVM	0.832	0.771	0.752	0.771
GBM	0.855	0.813	0.733	0.815
RF	0.838	0.82	0.72	0.822
XGB	0.845	0.753	0.789	0.752
KNN	0.730	0.899	0.553	0.907
Neural network	0.846	0.706	0.801	0.704

*AUC*, area under the curve; *LR*, logistic regression; *SVM*, support vector machine; *GBM*, gradient boosting machine; *RF*, random forest; *XGB*, extreme gradient boosting; *KNN*, *K*-nearest neighbors.

## Discussion

BC is a fatal urinary tumor that can be classified into non-muscle-invasive bladder cancer (NMIBC), muscle-invasive bladder cancer (MIBC), or clinical metastatic disease (19). The 5-year survival rate for mBC is only 5% (20). Patients with BCBM have the worst prognosis compared to other BM patients with urogenital cancers (21). The early identification of BM in BC could help improve the clinical outcomes. The available prediction methods have certain limitations. In this study, a GBM model was developed to assess the risk of BM in patients with BC. The model provides individualized risk stratification based on patient-specific characteristics (e.g., age, tumor stage, and histologic subtype), thereby informing personalized clinical decision-making. For patients across different risk categories, therapeutic strategies may be judiciously tailored—individuals at high risk might benefit from intensified multimodal regimens combining chemotherapy, immunotherapy, and targeted agents, while patients at low risk could potentially undergo reduced-frequency bone imaging surveillance—measures that may help alleviate financial burden, enhance quality of life, and mitigate metastasis-related complications.

Currently, the treatment strategies for BC are rapidly evolving. Immunotherapies and targeted therapies have transformed the treatment paradigm, offering broader and more effective therapeutic options for patients. Particularly noteworthy are the latest antibody–drug conjugates (ADCs), which have demonstrated significant benefits in BC (22, 23). The BM prediction model (GBM) developed in this study can provide decision-making support for ADC-based treatment strategies. For patients predicted to be at high risk of BM, we recommend direct adoption of combination therapy with ADCs and immune checkpoint inhibitors (ICIs). Studies have indicated that patients with metastatic predisposition who receive ADC+ICI combination therapy achieve a remarkable 1-year disease-free survival (DFS) rate of 97.4%, while the overall pathological downstaging rate reaches 75.5% (24), fully demonstrating the substantial advantage of this combined approach. AI is a research field that utilizes computers to simulate human intelligence, which has been successfully utilized in various domains, including autonomous driving, facial recognition, and music creation (25–27). ML, as a subset of AI, can assist clinicians in making better clinical decisions, thereby improving patient care and overall health (28). Tsai et al. (29) conducted a diagnostic study involving 1,336 patients with cystitis, BC, renal cancer, uterine cancer, and prostate cancer. The authors innovatively combined clinical laboratory data with ML methods to establish a diagnostic model for BC. Key indicators included calcium, alkaline phosphatase (ALP), albumin, urinary ketones, urethral occult blood, creatinine, alanine aminotransferase (ALT), and diabetes. Of the five models constructed in the study, LightGBM exhibited the best predictive performance, achieving an AUC value of 0.923 and an accuracy of 87.6%, demonstrating the potential of using clinical laboratory data for cancer detection. Xiong et al. (30) conducted a retrospective study involving 105 patients with BC. By comparing the performance of clinical models, radiomic models, and clinical–radiomic fusion models, the authors found that ML models combining radiomic features with clinical variables could more accurately predict the clinical staging of BC. Liosis et al. (31) developed an elastic net ML prediction model that successfully identified gene markers related to BC treatment response and disease progression, effectively predicting patients’ treatment responses and disease progression. Zheng et al. (32) created an ML algorithm based on pathological sections of MIBC to accurately quantify the tumor–stratum ratio (TSR) in patients. Their study showed a significant correlation between a low TSR and poorer overall survival, providing an automated TSR quantification method that reduces the subjectivity and inter-observer variability associated with traditional visual assessment methods. Despite significant progress in the construction and utilization of various models for the diagnosis, staging, treatment, and prediction of the prognosis of BC, there remains considerable room for improvement in the development of models that predict BCBM. For instance, Fan et al. (33) constructed a nomogram based on traditional logistic models to predict BCBM, identifying age, lung metastasis, liver metastasis, brain metastasis, N stage, T stage, histological type, pathological grading, primary tumor sites, and race as independent risk factors for BM in patients with BC. This study did not include patients’ previous treatment information, which could be considered in future model refinements. Zhang et al. (10) identified risk factors for BM in patients with BC, including age, race, marital status, T stage, N stage, tumor grading, lung metastasis, liver metastasis, and brain metastasis, but did not construct a corresponding predictive model.

In summary, while previous studies have developed nomogram models based on LR for predicting BM in patients with BC, these traditional approaches may have limitations in handling complex datasets. Our ML-based method offers an alternative approach that could potentially provide additional insights for clinical decision-making (34, 35). The existing prediction models for BCBM have shown varying performance. Identifying the risk factors for BCBM remains important for risk stratification and clinical management. In this study, ML algorithms were applied to analyze potential associations between clinical factors and BCBM risk, with the aim of developing an improved predictive approach (36).

Based on a big data analysis of the SEER database, this study identified independent risk factors related to BM through logistic regression analysis. A total of 12 clinically relevant variables associated with BCBM were included, namely, age, gender, race, tumor histology, tumor grade, T stage, N stage, radiotherapy, chemotherapy, brain metastasis, liver metastasis, and lung metastasis. Using multiple logistic regression analysis, 10 independent risk factors related to BM were identified: brain metastasis, lung metastasis, liver metastasis, radiotherapy, tumor grade, tumor histology, T stage, N stage, race, and age. BC exhibits diverse histological subtypes, including transitional cell carcinoma, squamous cell carcinoma, adenocarcinoma, and other subtypes. These variants demonstrated significant differences in biological behavior and prognostic outcomes (37). In this study, the limited number of BM-positive cases may have precluded comprehensive stratification to fully capture the heterogeneous impact of the histological subtypes on metastatic risk. Nevertheless, SHAP analysis confirmed their non-negligible contribution to the predictive model. Notably, chemotherapy was not identified as an independent risk factor for BM. This may be attributed to its predominant use in the advanced stage or in patients with mBC, who inherently exhibit a higher baseline risk of BM. Consequently, while chemotherapy appeared associated with BM in the univariable analysis, its effect became non-significant in the multivariable analysis after adjusting for T stage, N stage, and the presence of other metastases (e.g., liver/lung). These variables were incorporated into the model, enabling the development of an ML-based predictive tool. Model performance was assessed using standard metrics such as AUC, accuracy, sensitivity, and specificity on the test set. The GBM model demonstrated an AUC of 0.855, with a sensitivity of 0.733 and a specificity of 0.815, showing improved predictive capability compared with the other models developed in the study. These results suggest that this model may help identify patients with BC at an increased risk for BM. Furthermore, the subgroup analysis revealed diminished predictive performance of the model in two specific populations: black patients and those aged over 80 years. This observed reduction in accuracy may be attributable to data limitations and potential selection biases inherent in the study design. The GBM model, an ensemble learning algorithm, iteratively builds decision trees to correct prediction errors. Its ability to capture complex nonlinear relationships makes it highly effective for disease prognosis and risk stratification (38). Using the SHAP method, we determined that the T stage, the N stage, radiotherapy, age, lung metastasis, and liver metastasis are important predictors of BCBM. By comparing the characteristic rankings from the ML model with the SHAP analysis results, it was found that the T stage and the N stage consistently ranked as the top two features, indicating their significant contribution to model predictions. In addition, it was observed that four variables—radiotherapy, age, liver metastasis, and lung metastasis—ranked among the top six in importance across both methods, highlighting their value in predicting BCBM. Furthermore, it is noteworthy that radiotherapy emerged as a significant risk factor in the multifactor logistic regression analysis, with its importance ranking third in the SHAP graph, following T stage and N stage. This result may be related to the potential of radiotherapy to alter the tumor microenvironment and disrupt the normal synthesis and folding processes of the endoplasmic reticulum (ER) proteins, thereby promoting tumor aggressiveness and metastatic potential (39).

This study has several advantages. Firstly, an ML-based prediction model that can accurately predict BCBM was established, offering a more reliable alternative to traditional nomogram prediction models. Secondly, this research further explored the relationships among different independent high-risk factors, providing new directions for future clinical studies. Thirdly, for interpretability, SHAP values were used to show how each feature affected the predictions, helping to explain the model’s behavior. Finally, the generalizability of the model was independently evaluated using an external validation cohort, thereby mitigating potential performance overestimation due to data-splitting bias or overfitting.

However, this study does have certain limitations. Firstly, this large retrospective SEER-based study may introduce selection bias, particularly for the exclusion of patients due to missing data who might have a higher BM risk or unique clinical characteristics that the model failed to adequately learn, potentially compromising the prediction accuracy for these subgroups in clinical practice. Secondly, SEER lacks detailed treatment variables such as chemotherapy regimens and dosages, reducing the clinical prediction credibility and precluding treatment effect analysis. Future studies should integrate electronic health records (EHRs) with chemotherapy/radiotherapy planning systems. Thirdly, the established BC risk factors (i.e., smoking and occupational/environmental exposures) are unavailable in SEER and were thus excluded from the model, limiting the prediction accuracy. A fourth limitation is SEER’s hospital-reported diagnosis risk misclassification: BM may be underreported in asymptomatic patients without confirmatory imaging, while clinical–pathological T/N-staging discrepancies may exist. Fifthly, SEER does not track post-metastasis survival or SREs, which hinders assessment of whether early prediction improves outcomes. Although the reliability of the model was validated using AUC, accuracy, sensitivity, and specificity metrics and its generalizability was confirmed through external validation, its predictive capability remains limited and requires prospective clinical trial validation. Finally, the external validation dataset exhibits both class imbalance and geographic homogeneity (originating from a single region), resulting in performance fluctuations and predictive bias in the external cohort. Furthermore, disproportionate representation across subgroups may contribute to diminished predictive accuracy for specific demographic strata.

Today, with the rapid development of AI technology, the combination of AI with imaging omics plays a significant role in precision medicine (40) and is widely applied in the diagnosis, risk stratification, and treatment of various tumors, including BC, liver cancer, lung cancer, and parotid cancer (41–45). Overall, radiomics plays a significant role in the diagnosis, treatment, and prognosis of patients with BC, which enables timely interventions and thereby improves patients’ quality of life (46, 47). Future research plans include applying ML in conjunction with imaging omics to predict BCBM. We believe that, with the continued advancement of AI technology, ML will become increasingly prevalent in biomedical science, demonstrating substantial potential for clinical transformation and promising to significantly transform future medical practices (48–50).

### Clinical implementation and challenges

The GBM model developed in this study demonstrated good performance in predicting BM in patients with BC. We plan to implement this model as an interactive risk calculator in clinical practice, where patients’ clinical characteristics can be input after BC diagnosis to obtain a preliminary BM risk score (represented as a 0–1 value, e.g., 0.30 indicating 30% risk). Patients at high risk would be prioritized for imaging examinations to assist clinical decision-making (see the model card in the **Supplementary Material** for details). However, several potential barriers exist for clinical integration: Firstly, clinical data integration poses challenges due to fragmented data across different information systems with inconsistent formats and missing values, potentially compromising input data quality. Secondly, establishing a multidisciplinary team that involves clinicians, data scientists, and other experts is crucial to develop implementation strategies, determine risk thresholds, create clinical guidelines and workflows, and obtain regulatory approvals and ethical clearance. Thirdly, considering the severe consequences of BM and the healthcare cost-effectiveness, action thresholds should be established through cost–benefit analysis to minimize the expected costs based on model-predicted probabilities. In addition, clinicians accustomed to traditional approaches might exhibit skepticism toward the new model, questioning its reliability and perceiving it as interfering with clinical autonomy, while the complex algorithm and multiple input features may hinder interpretability and clinician trust. To enable developers, clinicians, regulatory agencies, and other stakeholders to quickly understand the model’s applicable scope and potential risks, we have created a model card (see **Supplementary Table S3**).

## Conclusion

In this study, we developed a ML model to predict BM in BC using 10 routinely available clinical features. Among the tested models, the GBM algorithm showed the highest predictive performance, including in the external validation cohort. These results suggest that the GBM model may aid in the clinical assessment of metastasis risk and inform treatment decisions.

## Data Availability

Publicly available datasets were analyzed in this study. This data can be found here: https://seer.cancer.gov/data-software/.
