# Supplementary material for: Ensemble machine learning models for predicting bone metastasis in bladder cancer
Source: Front Oncol. 2025 Sep 25;15:1653506. doi: 10.3389/fonc.2025.1653506 (PMC12509218; doi:10.3389/fonc.2025.1653506)
Supplement: Supplementary file 1 [file Table1.docx]

**Table 1**

**In this study, models were developed using the "caret" package in R, employing ten-fold cross-validation on the training dataset combined with grid search for hyperparameter optimization**

| model | model parameter |
| --- | --- |
| Logistic | - |
| SVM | sigma = 0.001,C = 0.09 |
| GBM  XGB | n.trees = 100, interaction.depth = 5,shrinkage = 0.1, n.minobsinnode = 30 |
| Neural network  KNN  RF | nrounds = 10,max_depth = 3,eta = 0.001,gamma= 0.5,colsample_bytree =0.5,min_child_weight=1,subsample = 0.6  size = 6,decay = 0.6  kmax = 12 ,distance = 1,kernel = "optimal"  mtry = 11 |

**Table 2**

| Model | AUC | Accuracy | Sensitivity | Specificity |
| --- | --- | --- | --- | --- |
| Logistic | 0.789 | 0.899 | 0.636 | 0.908 |
| SVM | 0.775 | 0.786 | 0.727 | 0.788 |
| GBM | 0.766 | 0.945 | 0.455 | 0.962 |
| RF | 0.764 | 0.48 | 0.909 | 0.465 |
| XGB | 0.765 | 0.789 | 0.727 | 0.791 |
| KNN | 0.671 | 0.804 | 0.636 | 0.81 |
| NeuralNetwork | 0.736 | 0.789 | 0.545 | 0.797 |

**Table 3**

**Model Card:**

| Core content |  |
| --- | --- |
| Basic information of the model | whether bone metastasis occurs |
|  | The input features include patient age, T stage, N stage, radiotherapy history and 10 other clinical variables. |
| training data  Performance  Ethics and Fairness  Recommendations for clinical use | The output is the probability of bone metastasis (ranging from 0 to 1).  The data was sourced from the SEER database (2010-2015, U.S. population).  The study included 22,114 cases, among which 537 (2.4%) had bone metastasis.  The study has limitations including missing data on smoking history and chemotherapy details.  The model achieved an AUC of 0.855 on the test set.  The model is intended for use with newly diagnosed bladder cancer patients, with specific exclusion criteria such as those with existing distant metastases.  Subgroup analyses are needed to evaluate performance differences across racial/ethnic and gender groups.  The findings may have potential biases as the data was derived solely from the U.S. population, which may limit generalizability to other geographic regions.  This tool is designed for auxiliary screening of high-risk patients and should be used in conjunction with imaging examinations.  The recommended risk threshold for triggering clinical intervention is a probability >0.7, though this should be adjusted based on subsequent validation studies.  This model is not intended for non-bladder cancer patients or pediatric populations. |
